# Treatment of Hemorrhage from the Bowels in Typhoid Fever

**Published:** 1872-08

**Authors:** S. Weed

**Affiliations:** Clyde, N. Y.


ART. IV.—*Treatment of Hemorrhage from the Bowels in Typhoid Fever.* S. WEED, Clyde, N. Y.

Read before the Central New York Medical Association.

In the present paper I do not propose to treat of the symptoms of typhod fever *in extensio*, nor of its morbid anatomy, pathology, diagnosis or prognosis, but merely to refer to a grave complication, which not unfrequently shows itself during the progress of this disease and the remedy, in my judgment, best adapted to meet the exigencies of the case or indications of treatment.

The grave complication, or symptom to which I refer, is that of hemorrhage from the bowels. Wood in his "Practice of Medicine," says that "this is often, at the same time, a bad sign, and injurious by the exhaustion it produces." Bartlett in his work on the "Fevers of the United States," page 124 speaks of it as "A grave symptom." "Of seven cases cited by Chaucel, all but one terminated unfavorably." "Of seven cases mentioned by Louis, three were fatal." And of thirty one in the Massachusetts General Hospital, eleven only terminated unfavorably."

Without quoting from or refering to other authorities, it may be safely affirmed, that this symptom, occurrring during the progress of an otherwise naturally exhaustive fever, is well calculated to excite alarm to the physician and among the friends of the sick. So far as my experience goes, it is not confined exclusively, as might be supposed, to cases of the greatest severity. It not unfrequently manifests itself in the latter stages of a fever, of otherwise, mild type. I have known it also, to occurr, during convalescence; the discharges of blood being very large and consequently alarmingly exhaustive. Two of the five cases, which I propose to report at this time, happened during this period; and if it had not been that I was on the look-out for this complication, owing to the condition of the bowels and a disposition to epistaxis, it seemed probable that, one or both of them, might have terminated fatally.

Now what treatment do the books advise us to pursue in an emergency in this kind? Various astringents, such as acetate of lead, kino, rhatany, tannic and gallic acids etc., combined with opium, or some of its preparations, are, I believe, usually recommended. But the operation of these therapeutic agents are, not always, satisfactory.

CASE 1.—In the evening of the 2nd. day of the month of Sept., 1852, I was requested to visit W——a boy of sixteen years, with typhoid fever, some two and a half miles distant, and who had been under the care of an other practitioner some two or more weeks. I was told that the case was one of great urgency, since, an unfavorable prognosis had been given; on arriving at the bed side, I was informed, that blood, in large quantities, was passing from his bowels at each frequent evacuation. Found patient exceedingly restless from pain and tympanitic distention of the bowels. Skin dry and burning, pulse extremely rapid and thready, tongue dry, clean and with dark papilla, with sordes on the teeth and lips. The prognosis, indeed, seemed most unfavorable.

In being called upon to prescribe in an energency of this kind, there was an imperative demand for immediate and dicisive action. What was to be done should be done quickly. Not a moment was to be lost. In running rapidly through my mind the various styptic remedies of the *materia medica,* suitable to the case before me, I happened to recollect an article first published in the *Medical Times* August 17th, 1850, from the pen of Dr. Wm. Budd, physician to the Bristol Infirmary, on the "styptic properties of oil of turpentine in a case of purpura hemorrhagica," and also another article in *Braithwaite's Retrospect* for January 1851, by John Griffith, Esq. Wiexham, on the use of turpentine, in large doses, in uterine hemorrhage; and from the high praise given this remedy by these medical gentlemen I at once, resolved to give it a trial. Some was procured from a near neighbor, and, without delay I administered a teaspoonful in some sugar and water, and in fifteen minutes as much more. After the expiration of an hour, I gave half the quantity in the same manner; and then ordered that in two hours twenty drops should be given and so on, every two hours, until I should see the patient again the following morning.

Sept. 3d. Symptoms much improved. Pulse slower and fuller. Less heat of surface, with a tendency to perspiration. Expression of countenance less anxious, and from the character of the stools, was fully convinced, that the turpentine had controlled the hemorrhage, almost immediately after the first dose had been taken. For the next twenty-four hours I ordered the remedy to be given in twenty drops doses every four hours.

Sept. 4th. Patient still improving. Symptoms all better. No more hemorrhage. But the turpentine was so obnoxious, that I reluctantly discontinued its further use until I should see him again, and substituted a tonic in its stead.

Sept. 5th. Hemorrhage had returned with symptoms of a very threatening character. I now prescribed the turpentine again, to be given in twenty drops doses, as first, every two hours for three or four doses depending upon symptoms, and then every four hour until I should see him next day. Without extending the report of this case farther, I will briefly state, than I continued the remedy some three or four days, at the expiration of this time, convalescence was fully established, and without further draw-back went on quite rapidly to complete recovery; there being no more hemorrhage.

CASE 2.—On November 13th, 1865, I was requested to visit M. a girl of eleven years, with typhoid fever, in great haste, and earlier than was my custom; and whom I had been visiting daily for some two weeks. She was represented to be dying from hemorrhage of the bowels. On arriving at the house I was told that the bed pan had once been emptied, and that the quantity of blood it contained was about the same as was then in it, which appeared to be not less than one pint. She was nearly pulseless, and had every appearance of one dying from loss of blood. Her mother was so strongly impressed with the conviction, that there was no hope, that it was with much difficulty I could persuade her to assist me in giving a teaspoonful of oil of turpentine, in mucilage, to her daughter. We however succeeded, and at the expiration of an hour repeated the dose. I then prescribed twenty drops to be taken every two hours until I should visit her again. Called in the evening and found symptoms improving. Was satisfied that the first dose of turpentine had controlled the hemorrhage as if by magic. Continued the remedy some three or four days, when convalescence being fully established it was discontinued. The recovery in this case was very satisfactory.

CASE 3.—Was that of C. a young man of about twenty-one years, living some four miles in the county, whom I attended in a long run of typhoid fever, of a severe type;—my visits extending from December 19, 1865, to January 13, 1866. There was much delirium and disturbance of the bowels during its progress. In the third week of his illness he had an attack of bleeding from the bowels which was very readily controlled by the use of the oil of turpentine. Patient recovered.

CASE 4.—On the evening of December 19, 1865, I was requested to visit Mr. B. of middle age, who resided in an adjoining town, and who had been under the care of a neighboring physician between two and three weeks. I found him, with the usual symptoms, of a mild type of continued fever. There was considerable debility for the amount of fever, night sweats and derangement of the bowels; but under the influence of tonics and stimulants, with suitable nourishment, he began to improve, until after having made nine visits, I ceased my calls, with the injunction, that should hemorrhage from the bowels show itself, at any time, I was to be, without delay notified. This was the only apprehension I had, respecting his recovery without a draw-back. We were having many cases of typhoid fever at this time, and many of them were fatal. The next evening a messenger came in great haste to let me know that Mr. B. had had a large evacuation of blood from the bowels, filling the chamber or vessel nearly, if not quite, half full. I prescribed the oil of turpentine as in the former cases; at first in teaspoonful doses, and then smaller ones as the urgency of the symptoms seemed to demand. The effect of the medicine was all that could be desired. The first dose operated to stop the hemorrhage. Patient recovered without any other unpleasant symptom.

Case 5.—Was that of F. a lad of seventeen summers, whom I attended, in an adjoining town, during a portion of the month of Nov. last. He had been ill some three or four weeks, with a mild from of typhoid fever. After treating him some ten days, he had so far recovered, as to make my visits no longer necessary. But from the disposition to epistaxis, and diarrhœa, with occasional traces of blood, in the fæcal evacuations, I gave directions that, should hemorrhage from the bowels occur at any time, during the period of convalescence, the oil of turpentine must be given without delay. In about a week, the father of the boy, called at my office and stated that the next day after my last visit, a pretty sharp hemorrhage of the bowels set in, for which they had given the remedy in half teaspoonful doses, with the happiest results; it having controlled the bleeding very speedily; and that convalescence was going on very satisfactorily.

To enter fully upon the therapeutics of the oil of turpentine, or history of its introduction into the materia medica, is not the object I have in view at this time. It would be too much of a trespass upon your time and patience, my sole purpose being to throw what light I can upon, or call your attention more especially to, the management of hemorrhage of the bowels, when it occurs as a complication in the latter stage of typhoid fever. It will be noted that the doses given are much larger than recommended by our works on materia medica in passive hemorrhages from the bowels. How the remedy acts, whether as a stimulant or styptic to an ulcerated surface, you can judge as well as myself. I am well aware that oil of turpentine has been much used of late years in the ordinary dose in certain stages, or to combat certain symptoms, of typhoid fever, but not in the hemorrhage occurring during their progress.

